# Supplementary figures and images for: Mutation in Wdr45 leads to early motor dysfunction and widespread aberrant axon terminals in a beta-propeller protein associated neurodegeneration (BPAN) patient-inspired mouse model
Source: Front Neurosci. 2025 Feb 28;19:1545004. doi: 10.3389/fnins.2025.1545004 (PMC11907653; doi:10.3389/fnins.2025.1545004)

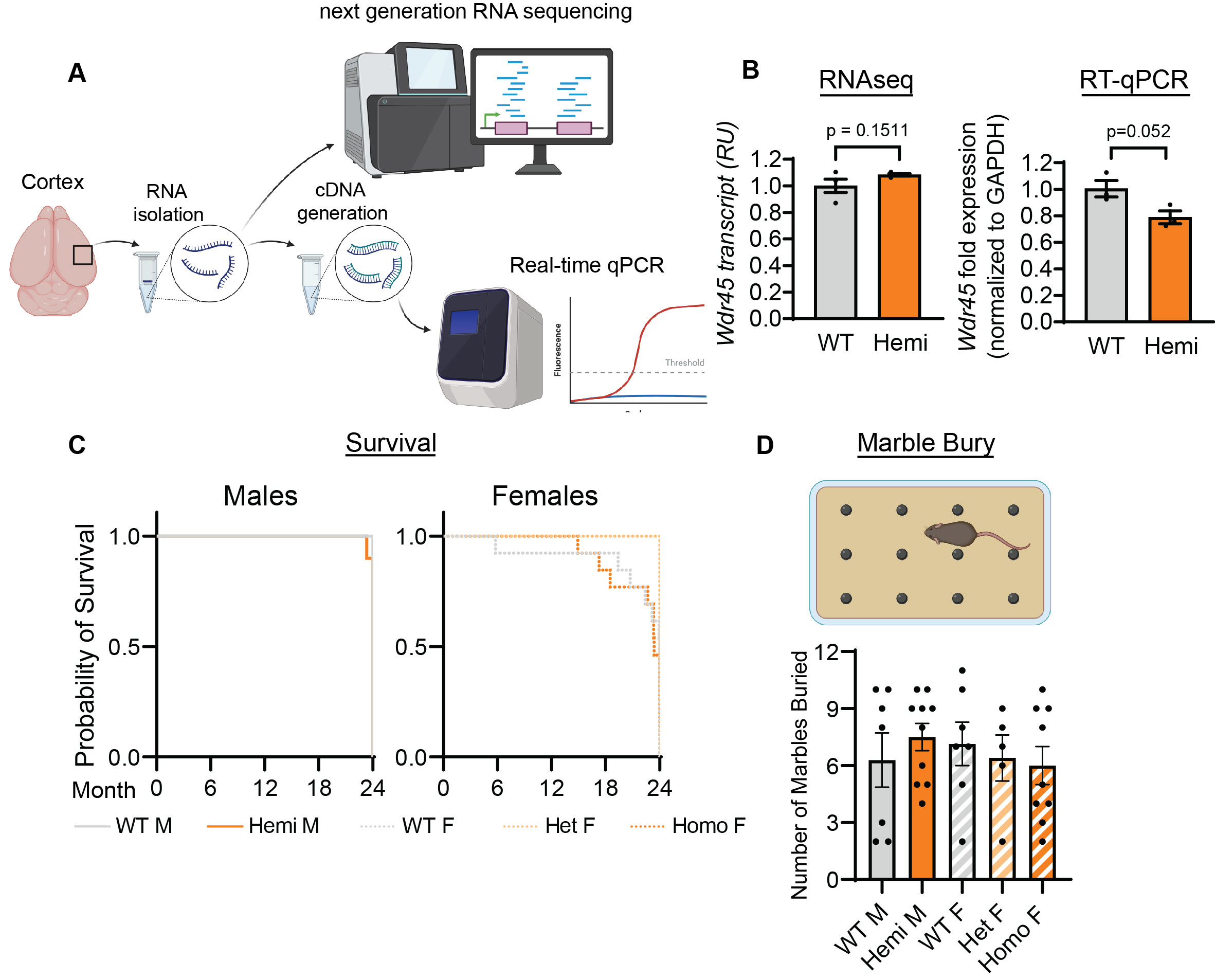

Supplement: Supplementary Figure S1 — Wdr45 c52C>T animals have unchanged Wdr45 transcript but exhibit unaltered survival and anxiety phenotypes. Wdr45 c52C>T animals have unchanged transcript abundance in the cortex at 3 months of age (B). Wdr45 c52C>T animals showed unchanged survival through two years of age (B). Additionally, no difference was seen between genotypes in the marble bury test performed at 3 months of age (D). For D: Testing was performed using Mantel Cox test on males and females separately. For B: Males were tested using a T-test. Females were tested using a one-way ANOVA with Holm-Šídák post hoc test. N = 8-14 per group. Mean ± SEM. [file Image_1.JPEG]

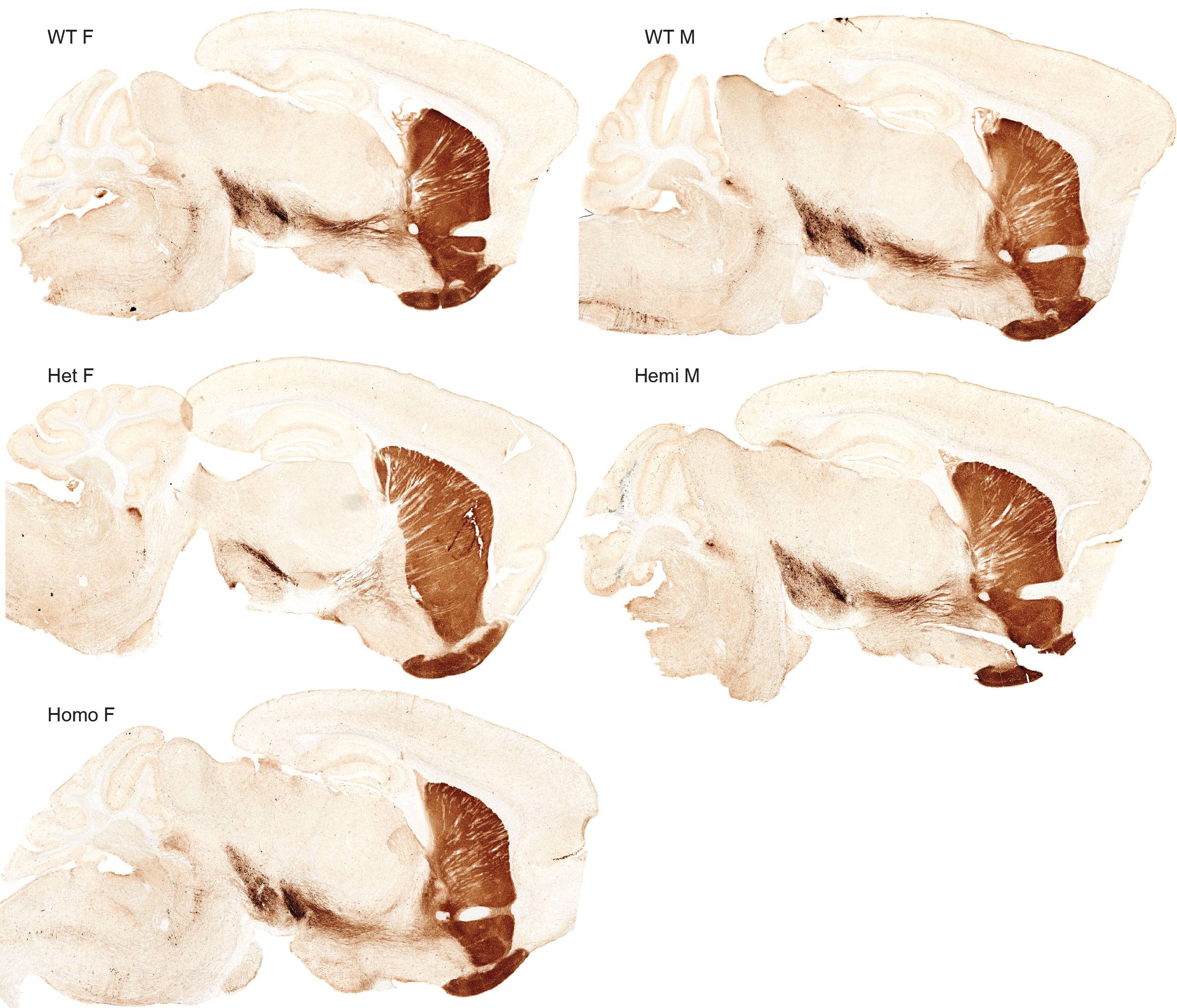

Supplement: Supplementary Figure S2 — TH positive spheroids appear throughout the brain of Wdr45 c52C>T mice at 3-months-of-age. Full sagittal section scans of immunolabeling against TH in 3-month Wdr45 c52C>T mice. [file Image_2.JPEG]

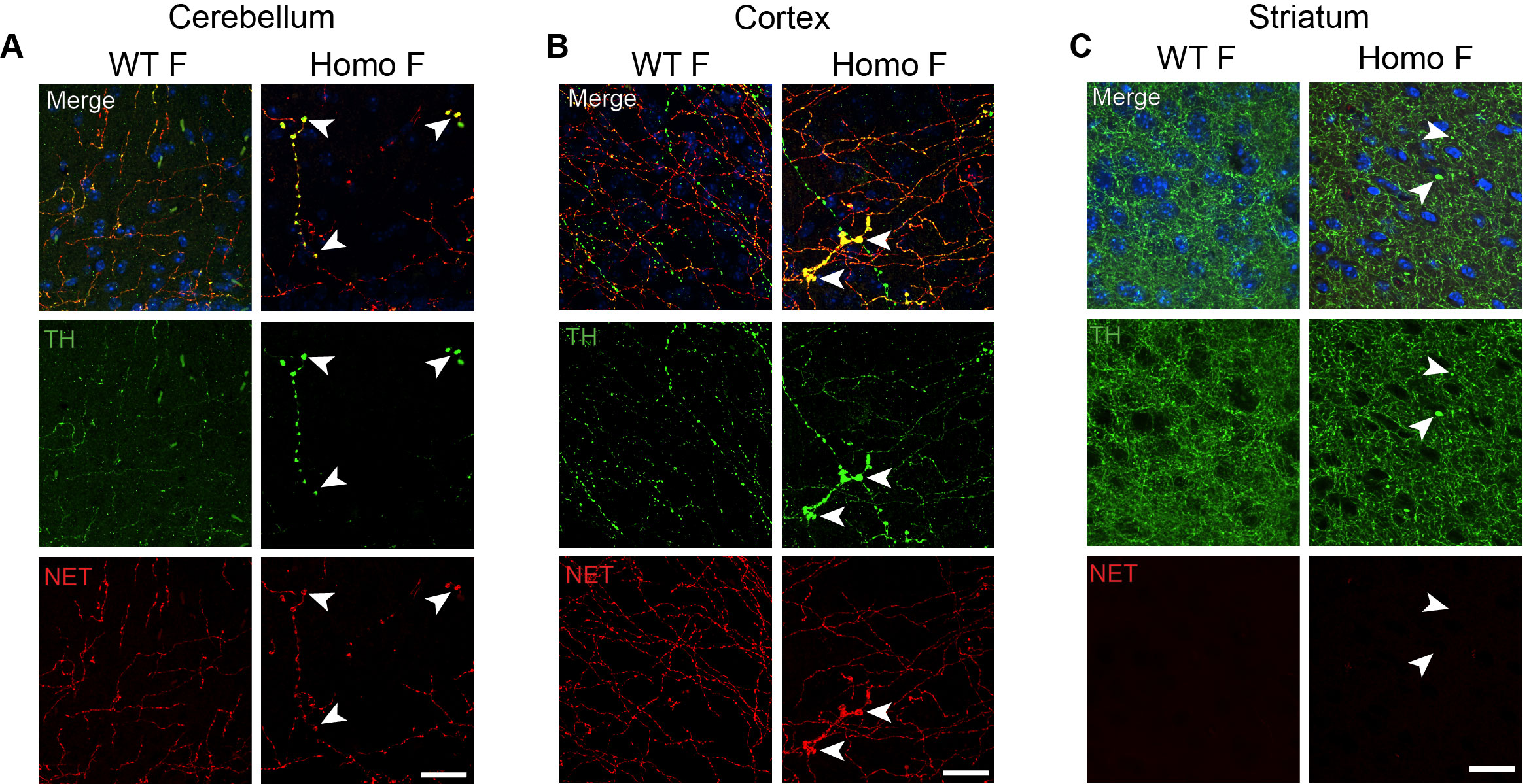

Supplement: Supplementary Figure S3 — TH+ spheroids are NET positive in cortex and cerebellum, though not the striatum. Immunofluorescent labeling of TH (Green) and colabeling of Norepinephrine Transporter (NET) (Red) indicates that these structures in the cortex and cerebellum are surrounded by this transmembrane transporter (A,B). Striatal TH positive spheroids do not show NET co-labeling. A,C scale bars = 25μm. [file Image_3.JPEG]

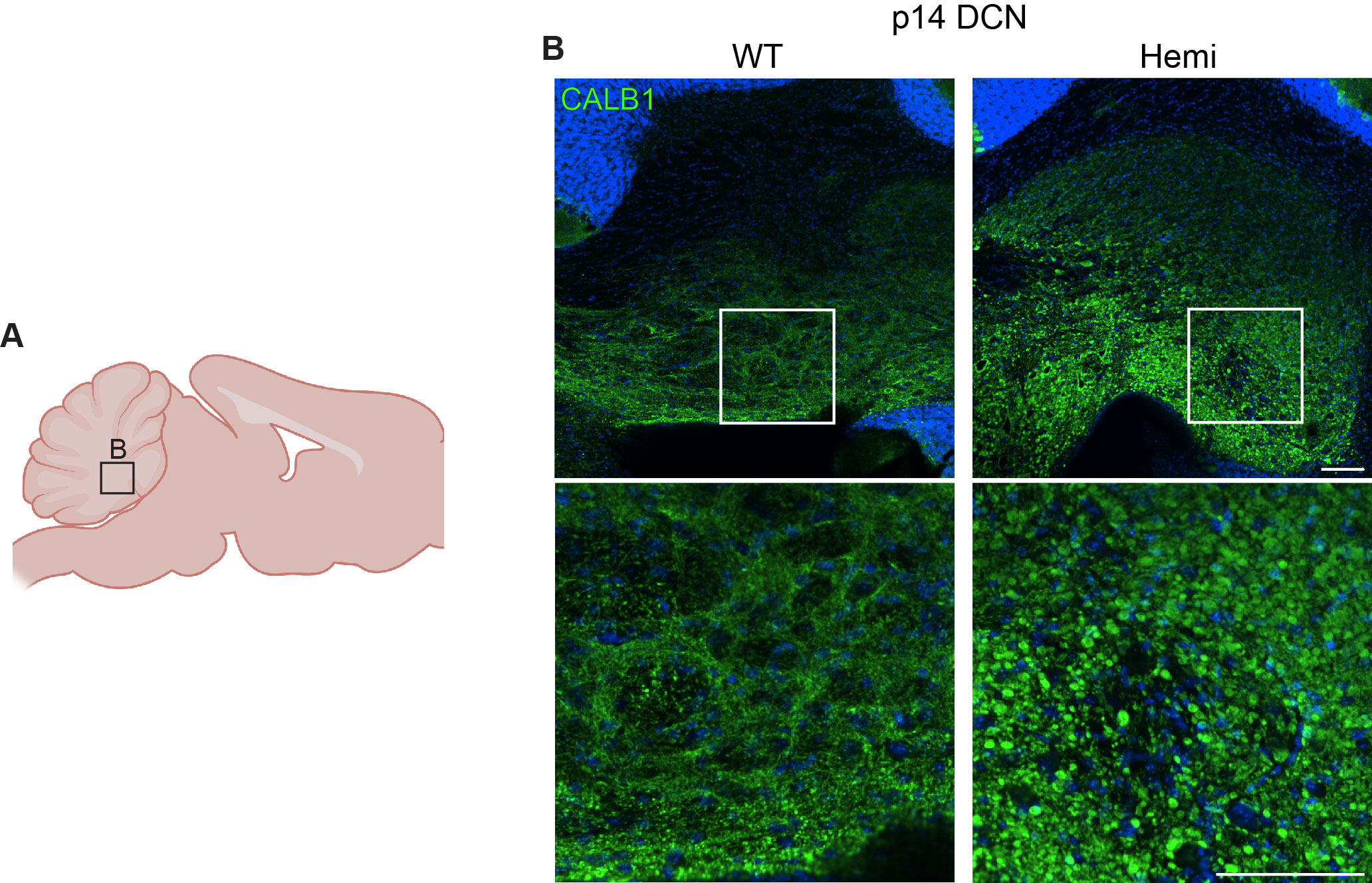

Supplement: Supplementary Figure S4 — Calbindin positive spheroids appear at p14. Diagram of imaged area (A). Calbindin (CALB1- green) positive spheroids appear in the DCN of Wdr45 c52C>T mice at post-natal day 14 (B). Scale bar = 100μm. [file Image_4.JPEG]

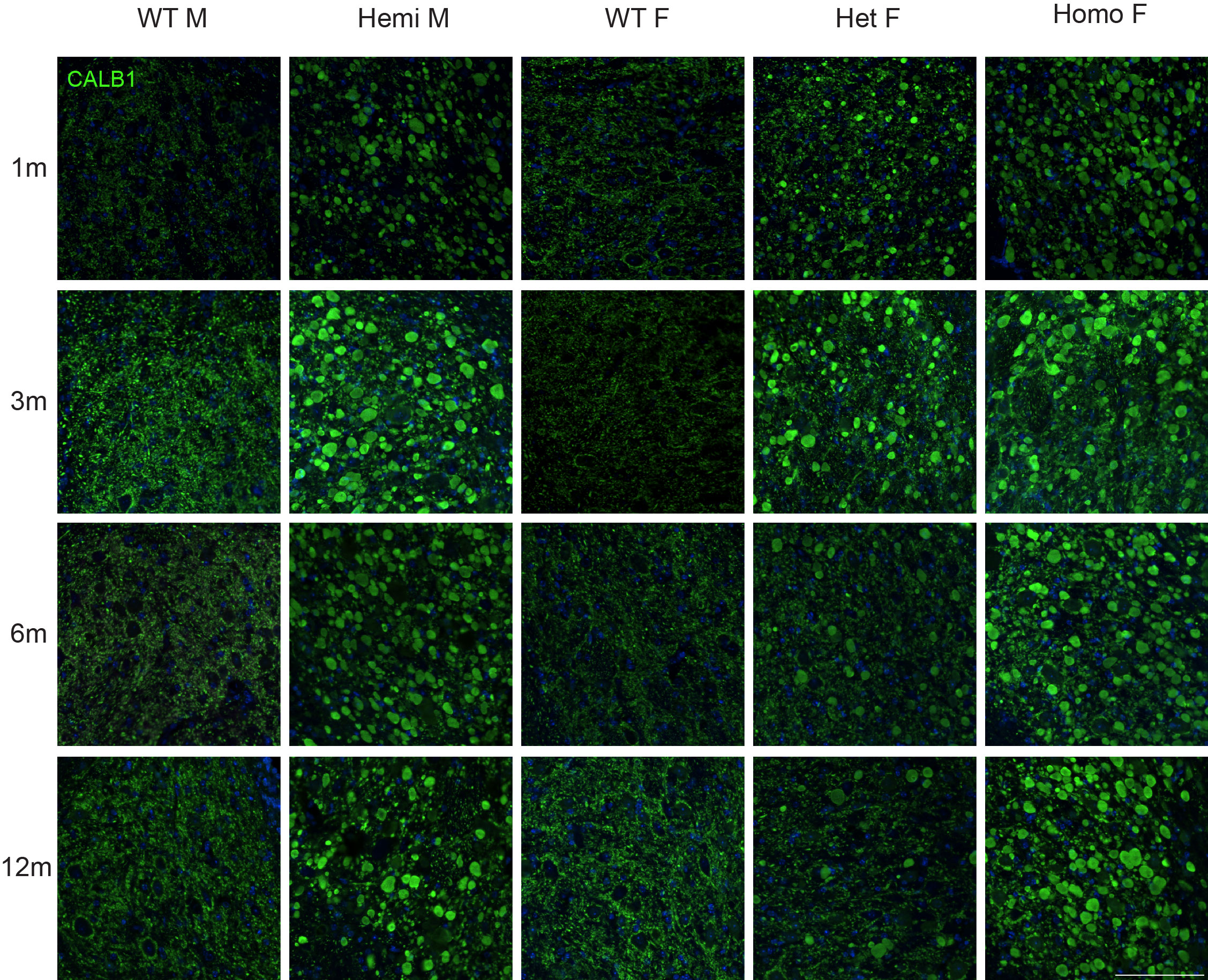

Supplement: Supplementary Figure S5 — Calbindin positive spheroids appear in Wdr45 c52C>T mice at all analyzed timepoints. Representative images of Calbindin labeling in the DCN of Wdr45 c52C>T mice. Scale bar = 100μm. [file Image_5.JPEG]

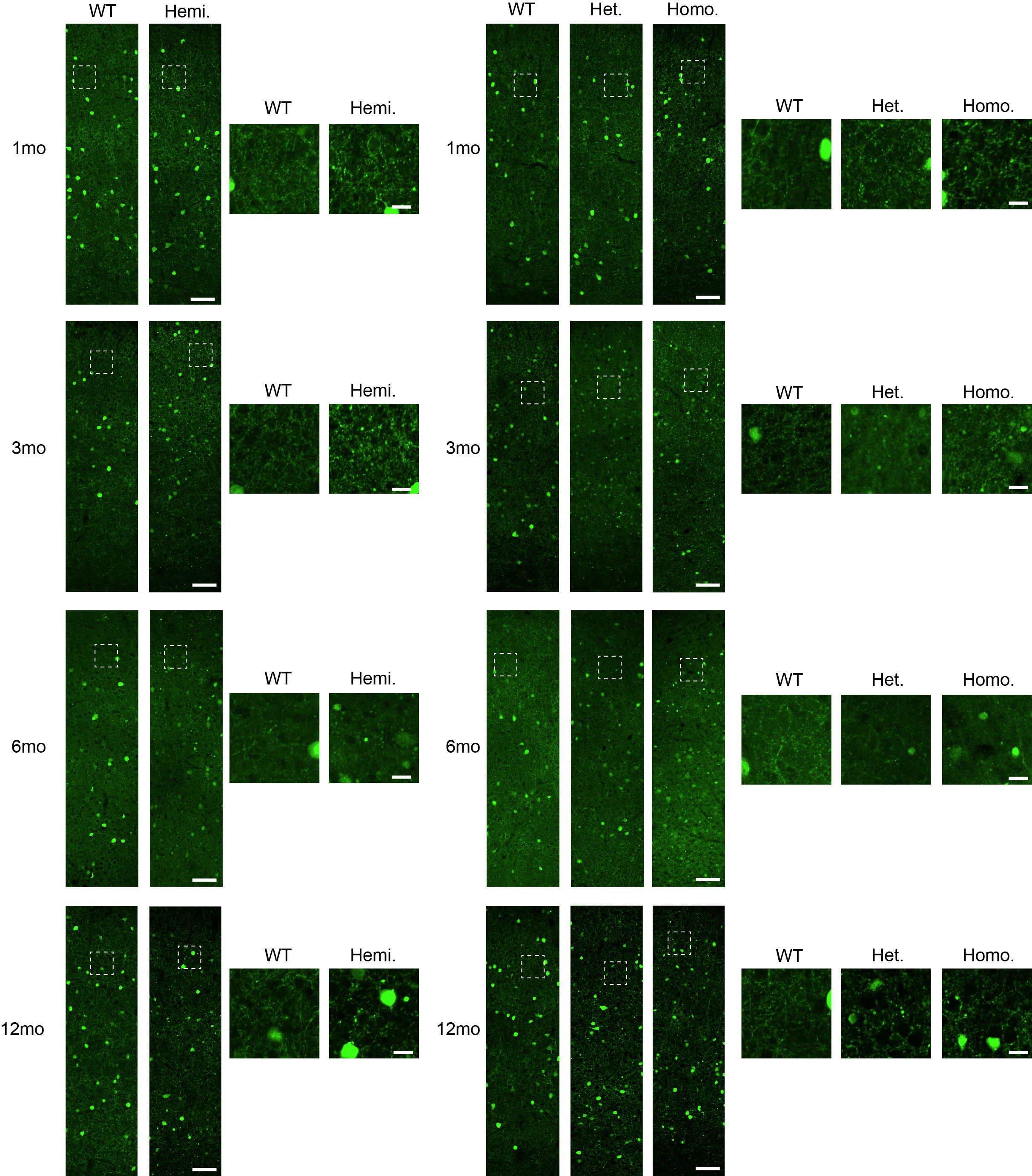

Supplement: Supplementary Figure S6 — Parvalbumin positive spheroids in the somatosensory cortex localize in distinct cortical regions compared to the cell bodies. Parvalbumin (PV) labeling in the cortex of 3-month-old animals (A,B) showed no difference in number of Parvalbumin positive cell bodies or layer location of these cells between genotypes (C). Comparison of PV positive cell bodies and spheroids. N = 3-4 per genotype. C,D used Two-way ANOVA with Holm-Šídák post hoc test, N = 3-4 animals for each group. Mean ± SEM: *p<0.05, **p<0.01, ***p<0.001, ****p<0.0001. [file Image_6.JPEG]

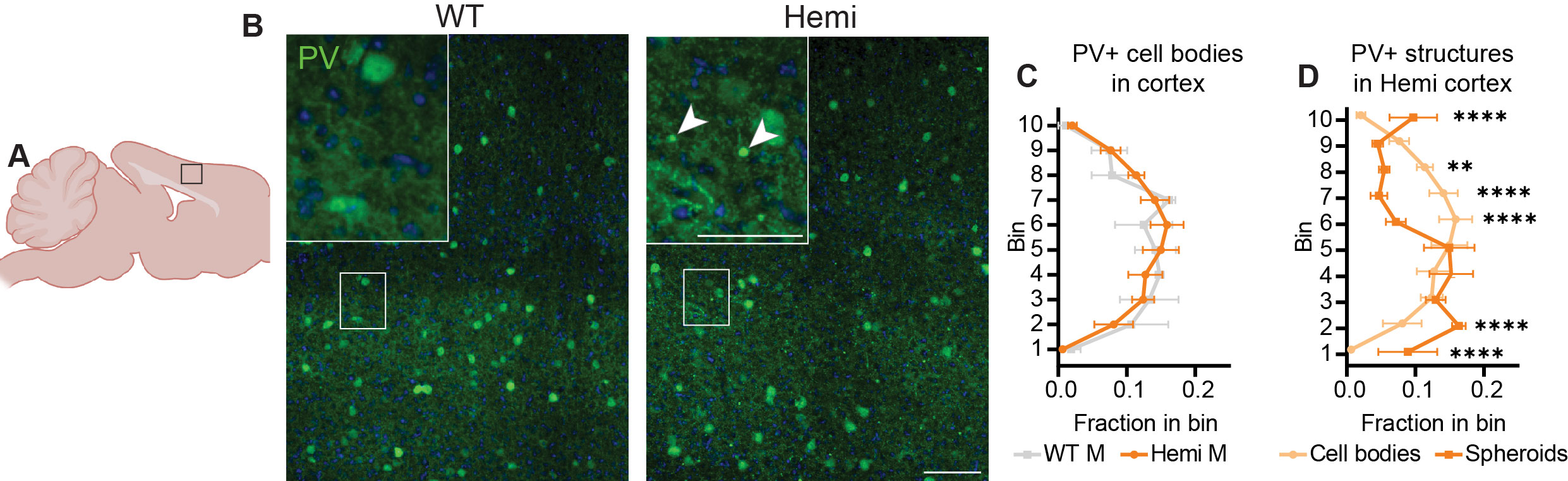

Supplement: Supplementary Figure S7 — Parvalbumin positive spheroids appear in Wdr45 c52C>T mice at all analyzed timepoints. Representative images of Parvalbumin labeling in the somatosensory cortex of Wdr45 c52C>T mice. Scale bars = 100μm, insets = 20μm. [file Image_7.JPEG]

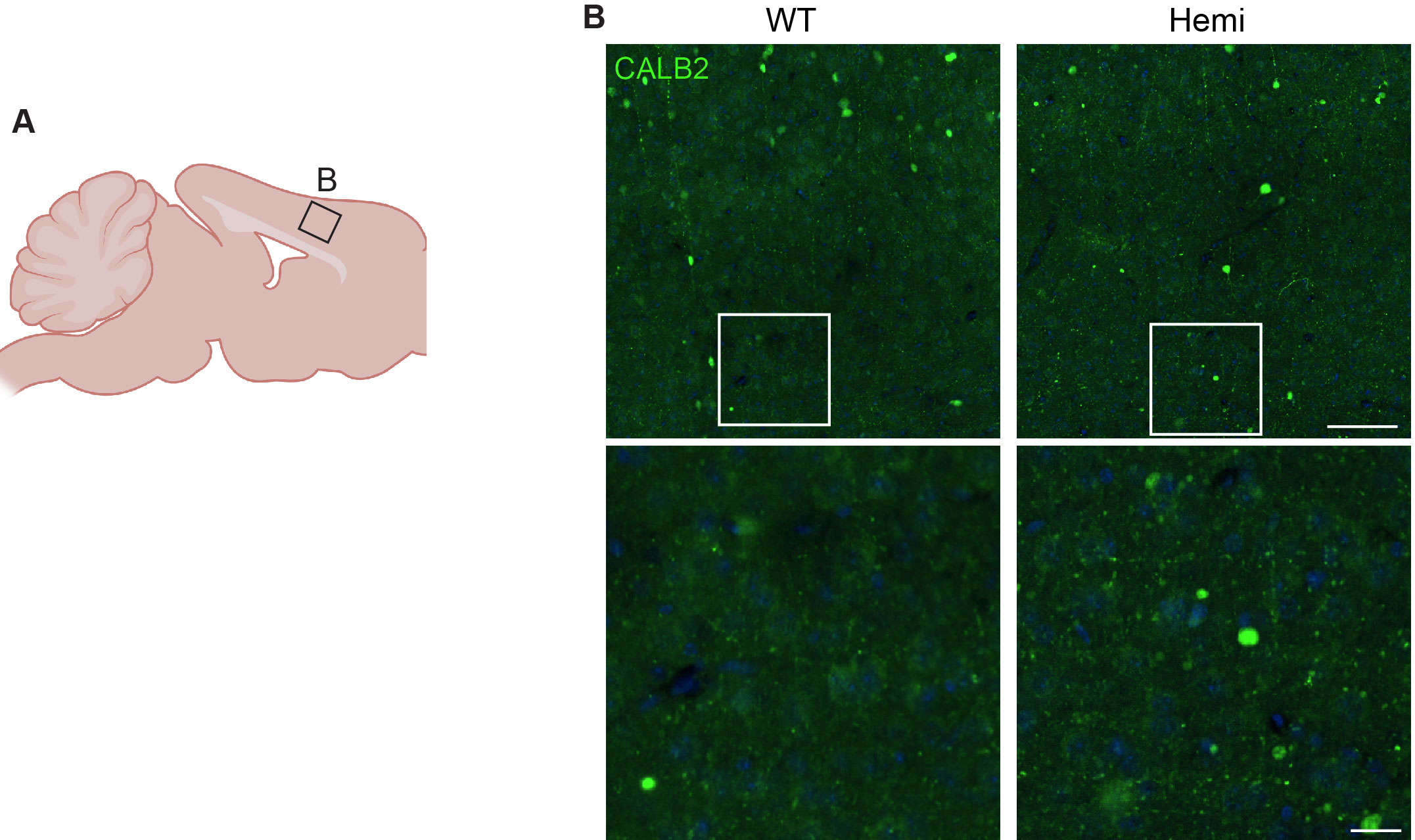

Supplement: Supplementary Figure S8 — Wdr45 c52C>T mice show CALB2 positive spheroids in the somatosensory cortex. Diagram of imaged area (A). Calretinin (CALB2- green) positive spheroids appear in the somatosensory cortex of Wdr45 c52C>T mice at 3-months (B). Scale bar = 100μm, inset = 20μm. [file Image_8.JPEG]

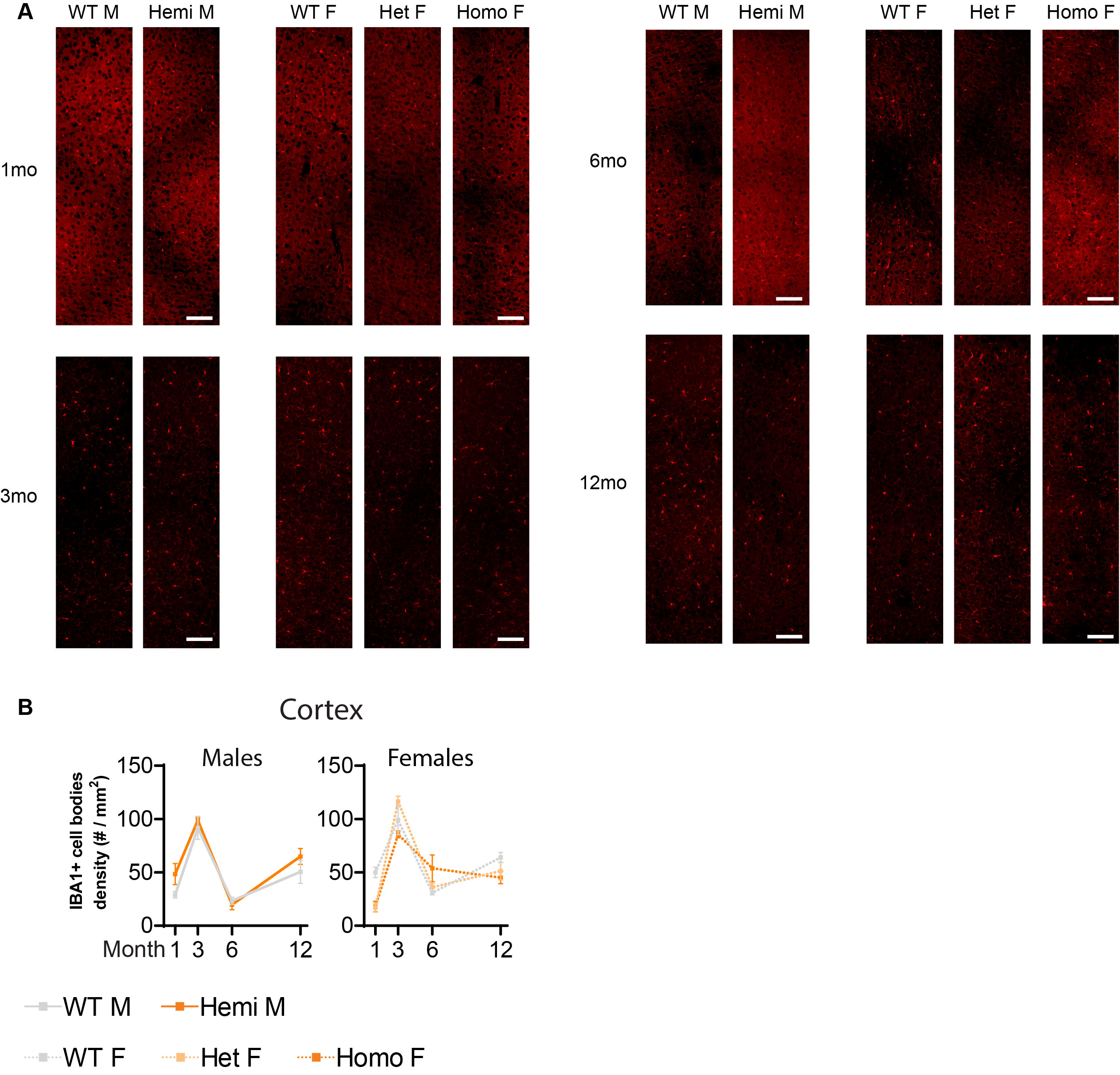

Supplement: Supplementary Figure S9 — Wdr45 c52C>T mice do not show increased number of microglia in the somatosensory cortex. Representative images of IBA1 immunolabeling over time in Wdr45 c52C>T mice (A). Number of IBA1 positive cells were not altered in the somatosensory cortex of Wdr45 c52C>T mice. B used Two-way ANOVA with Holm-Šídák post hoc test, N = 3-4 animals for each group. [file Image_9.JPEG]

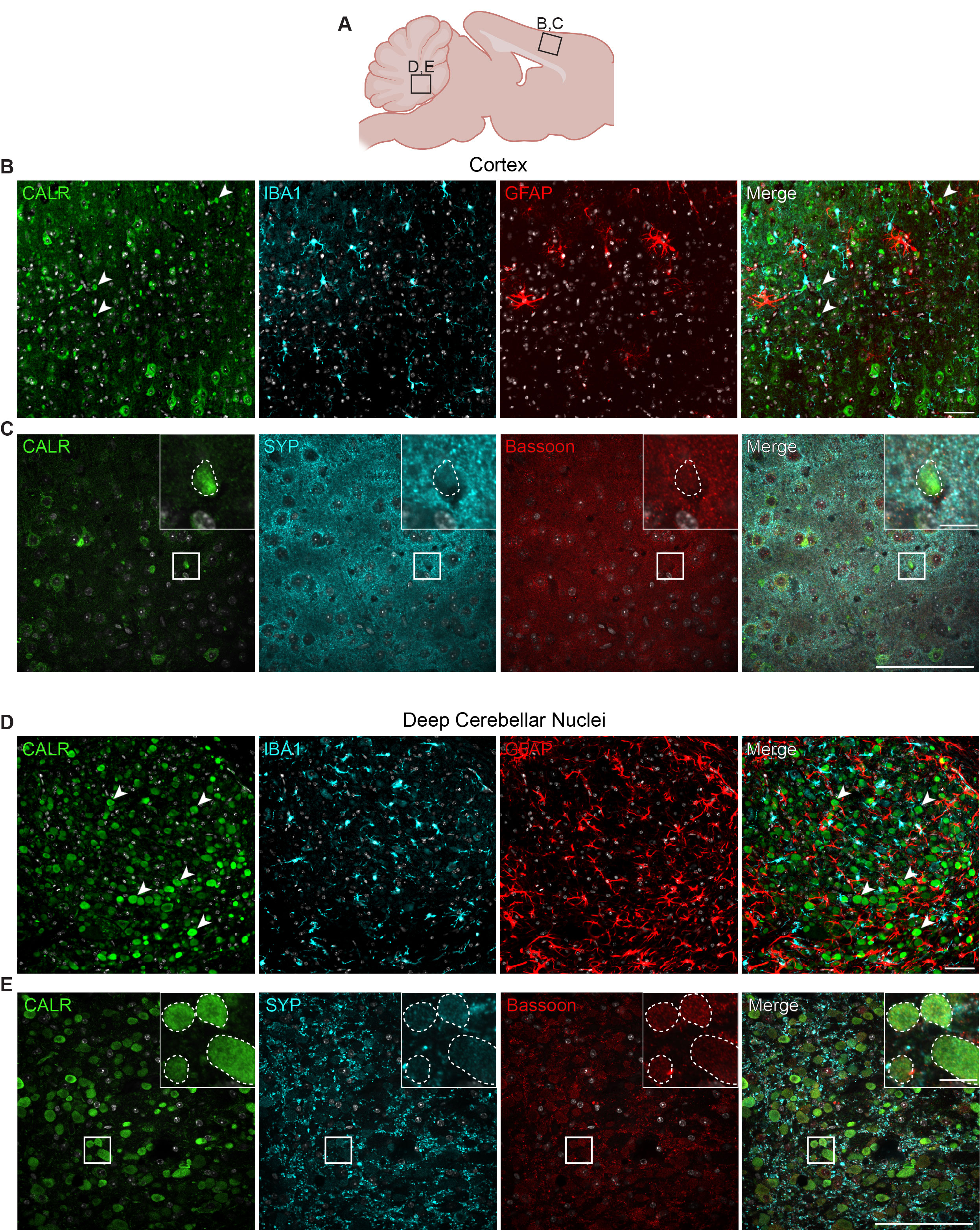

Supplement: Supplementary Figure S10 — CALR puncta are distinct from glial cells and positive for neuronal proteins in Wdr45 c52C>T mice. Diagram for areas imaged (A). CALR (Green) puncta do not colocalize with microglia (IBA1 - Cyan) or astrocyte (GFAP - Red) markers (B,D) but are positive for neuronal proteins (SYP-Cyan, Bassoon – Red) (C,E) at 6 months of age. Scale bars: B–D = 100μm, C,D inset = 10μm. [file Image_10.JPEG]
